# Rules and Regulations for Local Smallpox Quarantines

**Published:** 1899-10

**Authors:** W. F. Blunt

**Affiliations:** State Health Officer; Austin, Texas


### Rules and Regulations For Local Smallpox Quarantines.

The following instructions have been promulgated:

AUSTIN, TEXAS, August 31, 1899.

*To the County Health Officers of Texas.*

You will hereafter enforce the following rules and regulations in the management of local smallpox quarantines, together with such additional precautions as the interests of the public health may demand.

1st. On the discovery of smallpox in your county, immediately isolate the case or cases. If there is a doubt in the diagnosis, give the public health the benefit of that doubt. The diagnosis will soon develop itself.

2nd. Notify the commissioners' court of your county and ask them to declare quarantine.

3rd. Notify the State Health Officer, giving all the particulars, especially of the source of the contagion, if possible.

4th. On declaration of quarantine by commissioners' court, arrest and detain *all* persons who have been exposed.

5th. Locate a suitable pest house, remote from other houses and from public travel, and remove all cases to it.

6th. Locate a detention camp in the same manner, and remove all persons to it who have been exposed and hold them therein for eighteen days from date of last exposure.

7th. Sterilize all the clothing of those detained, either by boiling, soaking thoroughly in an *acid* solution of bichloride of mercury 1 to 500, or burn them.

8th. Disinfect the houses from which cases have been taken, by boiling all the clothing, bedding and other textile materials, washing down the walls with solution of 1 to 500 bichloride of mercury. Any furniture that cannot be boiled or *perfectly* cleaned by the solution shall be burned. Great care should be exercised to see that every part of the surface of these rooms and the furniture in them is thoroughly cleansed by the solution. The rooms should then be aired for five days when they may be occupied.

9th. The period of detention for those who have been exposed to the disease shall be eighteen full days from date of last exposure, and each person must be thoroughly examined and temperature taken before discharged.

10th. Those who have the disease should be held until desquamation ceases, then given a thorough bath with soap and water for two or three days in succession and discharged.

11th. No one, not even the guards, should be allowed to enter the detention camp without your permission. Should any one enter in violation of this rule, arrest and hold them in detention.

12th. Your guards should be reliable men, immune to the disease if possible, and will prove more zealous if selected from the immediate community you are engaged in protecting.

13th. The physician in charge should wear, when visiting cases of the disease, a long rubber coat, and immediately on leaving the sick room should wash his face and hands in a solution of bichloride 1 to 1000, and brush his outer clothing with brush or whisk-broom dipped in the same solution.

14th. Insist on the vaccination of every unvaccinated person in the community, especially insist on the vaccination and revaccination of those in detention camp.

15th. Make weekly reports of your operations to the State Health Officer.

W. F. BLUNT, M. D.
State Health Officer.

---

AUSTIN, TEXAS, September 9, 1899.

*Hon............................County Judge.*

DEAR SIR: There having occurred some conflict of opinion as to whose duty it is, under the law, to declare, maintain and pay for local quarantine, I have requested the Honorable T. S. Smith, Attorney-General of Texas, to render an opinion on this question and have received from him the enclosed opinion which is clear.

There is, however, one defect in the law that may, and frequently does, cause such delay in its execution as to result in the spread of infectious diseases, and greatly increases the cost of controlling them. The law does not empower the county health officer to take any steps in the isolation of infectious disease, or those exposed to the same, until quarantine has been declared by the commissioners' court. As it is frequently impossible to obtain a session of the court immediately, I would, therefore, suggest that it would be a wise plan to empower your county health officer to declare a temporary quarantine, and enforce the same, until such time as the court can be convened to take action. I am,

Very respectfully,

W. F. Blunt, M. D.,
State Health Officer.

